# Perigraft abscess after DeBakey type-1 aortic dissection: a case report

**DOI:** 10.1186/s13019-020-01128-9

**Published:** 2020-05-13

**Authors:** Lingling Xu, Shan Lin, Yali Yang

**Affiliations:** 1 Department of Ultrasound, Union Hospital, Tongji Medical College, Huazhong University of Science and Technology, Wuhan, 430022 China; 2 Hubei Province Key Laboratory of Molecular Imaging, 1277 Jiefang Ave, Wuhan, 430022 China

**Keywords:** Aortic abscess, DeBakey type-1 aortic dissection, Elephant trunk, Prosthetic vascular graft infection

## Abstract

**Background:**

Perigraft abscess is a rare condition which constitutes a small proportion of aortic graft infection (AGI). Early diagnosis is very important for timely intervention and improving the survival rate of patients because of its significant morbidity and mortality.

**Case presentation:**

A 24-year-old young male patient with a history of complicated total arch replacement using elephant trunk technique for acute DeBakey type-1 aortic dissection 6 months before visited our hospital with the chief complaint of persistent fever. Antibiotic treatment in local hospital was ineffective. Echocardiography showed liquid dark area around the aortic graft, and a computerized tomography angiography (CTA) was done for further evaluation of periaortic fluid collection which showed findings to suggest perigraft abscess. The patient underwent surgical debridement of the abscess and was found to have an abscess around the aortic graft which was drained followed by antibiotic treatment. The patient was discharged to his local hospital and recovered well at 2 month follow-up appointment.

**Conclusion:**

This is a very rare case of aortic abscess around the graft that could successfully be managed by graft-conserving surgery, and it emphasizes the significance of early diagnosis of perigraft abscess in patients with aortic dissection surgery.

## Background

AGI are rare events with an incidence of about 3% [1], and perigraft abscess is dreaded complications following aortic repair surgeries with high mortality rates. Early diagnosis is very important for timely intervention and improving the survival rate of patients because of its significant morbidity and mortality [2]. Here we report a rare case of perigraft abscess 6 months after complex aortic dissection operation.

## Case presentation

A 24-year-old young man was admitted to our hospital with chief complaints of high-grade fever more than 1 week. Antibiotic treatment in local hospital was ineffective. He denied a history of hypertension or a family history of cardiac disease. Six months prior to admission, the patient developed acute aortic dissection DeBakey type-1 for which he underwent complex surgery including removing autologous aortic walls of the ascending aorta and arch, David procedure, elephant trunk stenting and dacron graft repair of the total arch.

**Fig. 1:**
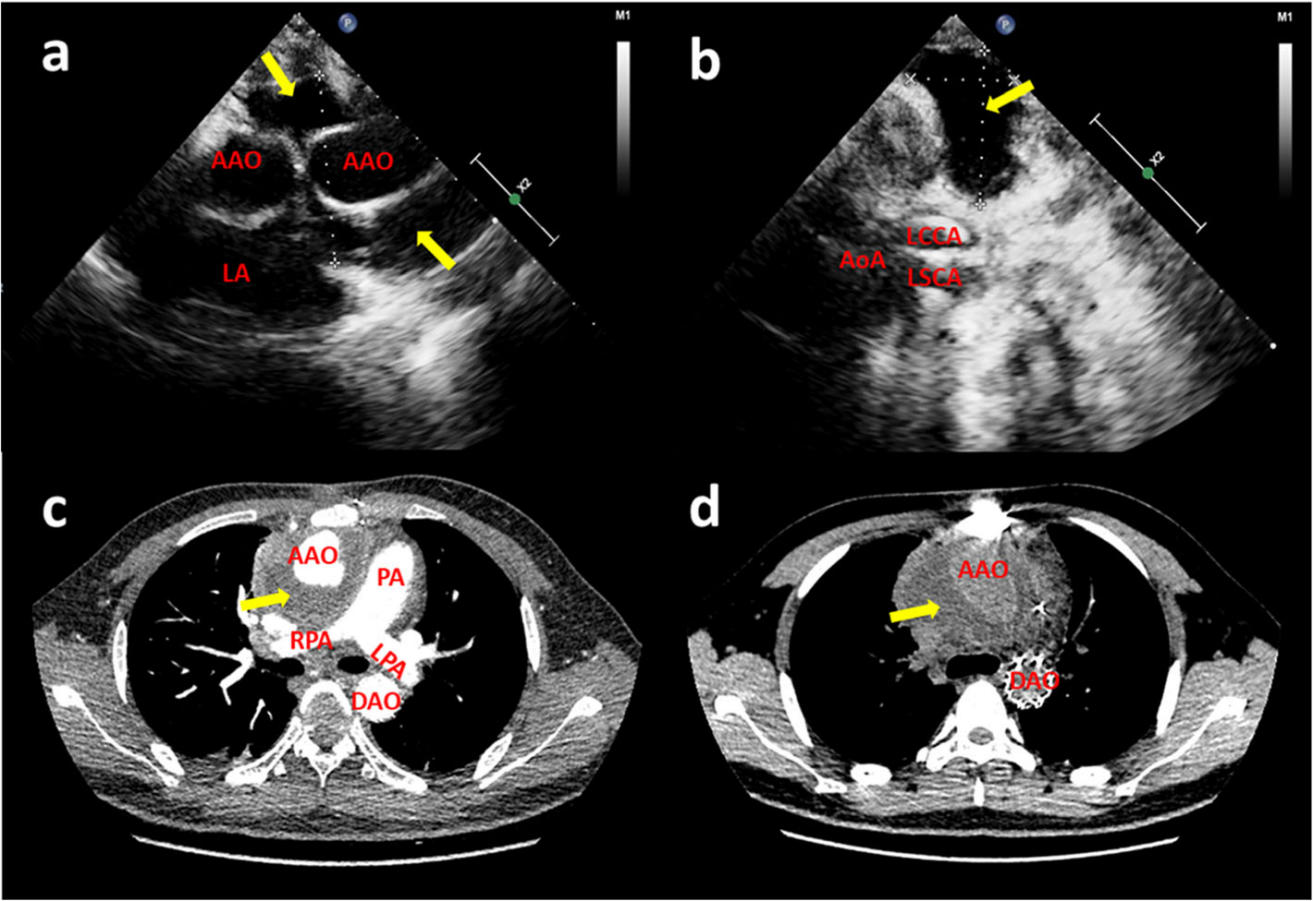
(**a**) Transthoracic echocardiography revealed liquid dark area and a little thrombus around the artificial blood vessels (yellow arrow); (**b**) The suprasternal view revealed a liquid area of the abscess (yellow arrow); CT angiography scan (**c** and **d**) showed perigraft liquid with low density around the artificial blood vessels (yellow arrow); Intraoperative photographs showed (AAO: ascending aorta; AoA:aortic arch; DAO: descending aorta; LA: left atrium; LCCA: left common carotid artery; LPA: pulmonary artery; LSCA: left subclavian artery; PA: pulmonary artery; RPA: right pulmonary artery)

On admission, his blood pressure was 104/84 mmHg, pulse rate 96 beats per minute, and respiratory rate 19 per minute. He was febrile with temperature of 37.8 °C. There was a swelling in the superior sternum fossa, the skin temperature was higher than that of the surrounding normal skin, and the yellow turbid pus flowed out from the surface breach. Hematological findings included C-reactive protein of 59.8 mg/L (reference range<7 mg/L), hemoglobin of 102 g/L (reference range from 120 to160g/L), white blood cell count of 23.35 G/L (reference range from 4 to 10 G/L). Blood bacterial culture was negative. Transthoracic echocardiography showed (Fig. 1a) an echolucent area consistent with fluid or blood around the ascending aorta conduit graft concerning for perigraft leak or abscess, but the valves were not involved. Liquid dark area was found in the subcutaneous tissue above the sternum with a few flocculent echo (Fig. 1b). Due to the concerning findings, a total aortic arch CTA scan with contrast was ordered and showed that the lesion surrounded the ascending aortic artery, aortic arch and its branches, extending into the level of the C7 vertebral body and going down to the origin of the aortic body (Fig. 1c;1d).

**Fig. 2:**
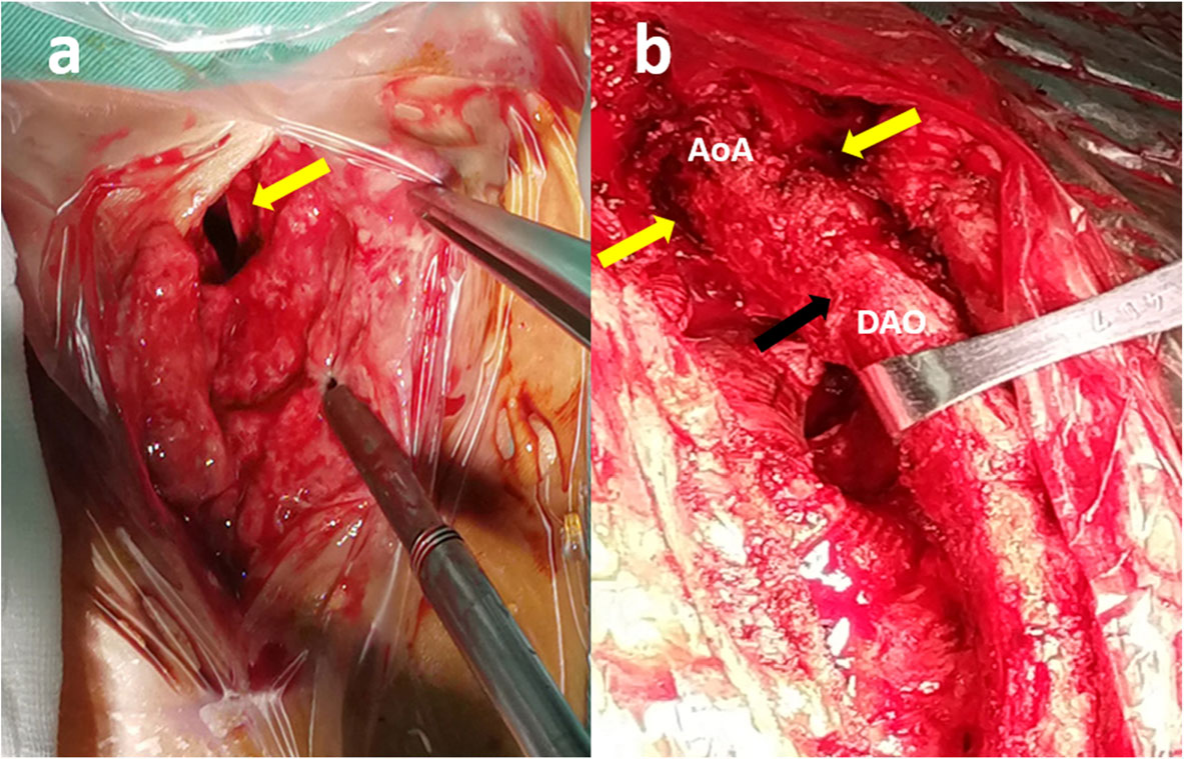
Median sternotomy: (**a**) Abscess cavity under the suprasternal fossa (yellow arrow), the pus had been cleaned up; (**b**) the shape of the artificial blood vessel was normal (black arrow), and the pus cavity could be seen around it (yellow arrows). (AoA:aortic arch; DAO: descending aorta)

This case was diagnosed with perigraft abscess and treated surgically. We chose a median sternotomy and saw the abscess cavity when opening the skin of the suprasternal (Fig. 2a). After thoracotomy, the old thrombus was cleared, and then washed repeatedly with active iodine and warm saline. No obvious bleeding was found. On surgical exploration, purulent material was drained from the wall of the aorta around the graft and microbiological cultures were obtained. The graft were normal in shape with a little old thrombus tissue (Fig. 2b). Intraoperatively, it was found that there was a clear demarcation between the graft and the abscess collection, and therefore, a decision was made not to explant the graft. After thoracotomy, the thrombus was cleared, then washed repeatedly with active iodine. Bacterial culture of pus and tissues collected during the surgery was negative. All surveillance culture samples collected perioperatively were also negative. Patient was still continued on intravenous antibiotics (Vancomycin mainly) for 2 weeks from the day after surgery. It based on the empirical use of antimicrobial agents in the treatment of infections. He started to defervesce postoperatively and remained afebrile upon discharge. As of 3 months postoperatively, there has been no recurrence of the infection.

## Discussion

Due to the rarity of AGI, there was no large number of studies to determine the susceptible factors of the disease, early postoperative infection was largely caused by direct microbial infection, and late infection mostly related to underlying diseases, postoperative internal leakage and thrombosis [3]. It is important to identify infectious pathogens in cases of prosthetic graft infection. The common pathogenic microorganisms were bacteria, especially staphylococcus aureus [4]. Late-onset AGI are usually associated with less virulent organisms such as coagulase-negative Stapylococcus, Cirynebacterium, or Propionibacterium species [5]. The bacterial culture of pus in this case was negative, which may be related to the prolonged antibiotic treatment before the examination.

Early diagnosis of AGI was difficult because of its nonspecific manifestations, and the mortality can be as high as 18% ~ 20% [6]. In 2016, the Britain Management of Aortic Graft Infection Collaboration (MAGIC) proposed the diagnostic criteria for AGI [7]. The preoperative radiology examination of this case met one of the major criteria (perigraft fluid on CT scan 3 months after insertion); the clinical manifestations met two minor criteria (localized clinical features of AGI e.g. erythema, warmth, swelling, purulent discharge, pain; fever≥38°Cwith AGI as most likely cause). Therefore, AGI was diagnosed before operation and the extent of involvement was accurately judged, which provided a reliable basis for clinical intervention in time.

The surgical management of AGI depended on the degree and extent of infection. It was generally believed that if the graft and autologous tissue were still surrounded by healthy autologous tissue, the survival rate was higher if the vascular prosthesis not removed [8, 9]. For this case, the graft was not infected and existing prosthetic grafts could be salvaged, so only debridement was performed and the vascular graft not replaced.

According to an international survey which incorporates 62 reported cases from different international centers of vascular surgery, mortality among operative cases was 16.3% and that among conservative management was 36.4% [10]. With such high mortality rates, it is important to focus on prevention, early detection, and prompt treatment of AGI.

## Conclusion

We experienced a possibly very rare case of late perigraft infection with aortic abscess formation around the prosthetic vascular. CTA and echocardiography are diagnostic modality of choice which can reveal perigraft fluid and loss of normal tissue. Debridement and drainage are accepted practice in anyone who does not have contraindications for surgery. If the graft is not infected by the abscess, it may be retained.

## Data Availability

Please contact the corresponding author for data on reasonable request.

## Abbreviations

AGI: Aortic graft infection
CTA: Computerized tomography angiography
AAO: Ascending aorta
AoA: Aortic arch
DAO: Descending aorta
LA: Left atrium
LCCA: Left common carotid artery
LPA: Pulmonary artery
LSCA: Left subclavian artery
PA: Pulmonary artery
RPA: Right pulmonary artery
